# Short and Long-Term Analysis and Comparison of Neurodegeneration and Inflammatory Cell Response in the Ipsilateral and Contralateral Hemisphere of the Neonatal Mouse Brain after Hypoxia/Ischemia

**DOI:** 10.1155/2012/781512

**Published:** 2012-06-03

**Authors:** Kalpana Shrivastava, Mariela Chertoff, Gemma Llovera, Mireia Recasens, Laia Acarin

**Affiliations:** Unitat d'Histologia Mèdica, Institut de Neurociències and Departament Biologia Cel.lular, Fisiologia i Immunologia, Universitat Autònoma Barcelona, 08193 Bellaterra, Spain

## Abstract

Understanding the evolution of neonatal hypoxic/ischemic is essential for novel neuroprotective approaches. We describe the neuropathology and glial/inflammatory response, from 3 hours to 100 days, after carotid occlusion and hypoxia (8% O_2_, 55 minutes) to the C57/BL6 P7 mouse. Massive tissue injury and atrophy in the ipsilateral (IL) hippocampus, corpus callosum, and caudate-putamen are consistently shown. Astrogliosis peaks at 14 days, but glial scar is still evident at day 100. Microgliosis peaks at 3–7 days and decreases by day 14. Both glial responses start at 3 hours in the corpus callosum and hippocampal fissure, to progressively cover the degenerating CA field. Neutrophils increase in the ventricles and hippocampal vasculature, showing also parenchymal extravasation at 7 days. Remarkably, delayed milder atrophy is also seen in the contralateral (CL) hippocampus and corpus callosum, areas showing astrogliosis and microgliosis during the first 72 hours. This detailed and long-term cellular response characterization of the ipsilateral and contralateral hemisphere after H/I may help in the design of better therapeutic strategies.

## 1. Introduction

With the improvement of perinatal care, the frequency of infant death has reduced considerably, but the incidence of neurological disabilities related to perinatal brain damage has not decreased in Western countries over the last decades [1–3]. Perinatal brain injury due to asphyxia, cerebral ischemia, cerebral hemorrhage, or intrauterine infection is the major contributor to perinatal morbidity and mortality as the immature brain is highly susceptible to damage. Injury to the newborn during the perinatal stage is the underlying etiology for a host of developmental disabilities that includes spastic motor deficits such as cerebral palsy [4, 5] and cognitive, behavioral, attentional, socialization and learning difficulties [6–9]. As brain development substantially influences the progression and hallmarks of brain injury [10, 11], it is not possible to apply therapeutic procedures used for adult ischemia to newborns.

 In term newborn infants, hypoxic/ischemic (H/I) brain injury is the most common cause of encephalopathy and seizures. Presently, optimal management of H/I brain injury involves prompt resuscitation, careful supportive care, and treatment of seizures. Although hypothermia is a promising new therapy, and recent studies suggested that head or whole-body cooling administered within 6 hours of birth reduces the incidence of death or moderate/severe disability at 12 to 22 months [12], there is undeniable need for the identification of new therapeutic targets for the implementation of clinical trials to address treatment of H/I encephalopathy [13]. Accordingly, epidemiological and experimental data have allowed researchers to identify a number of potential targets for neuroprotective strategies. Animal models have led to the elucidation of biochemical events involved in neurodegeneration and neuroprotection [14–18]; however, important differences among species have been described [19, 20].

 The initiation and development of injury to the neonatal brain is complex, with multiple contributing mechanisms and pathways resulting in both early and delayed injury [21]. As in other types of acute central nervous system (CNS) injuries, tissue damage and neurodegeneration initiate a cascade of inflammatory response depending on the nature and extent of damage, which is characterized by the involvement of damaged neurons, microglial, astrocytes, endothelial cells, and recruited blood leukocytes [22–25]. Microglial cells are the main nervous component of the innate immune system, playing a key role in the phagocytosis of cell debris to repair damage and maintain tissue homeostasis, but active producers of inflammatory mediators [26]. Astrocytes rapidly respond to extracellular changes and are the main cell type responsible for the restoration of blood-brain barrier, new glia limitans formation, and the establishment of a long-term glial scar [27]. In addition, vascular damage induces massive influx of blood leukocytes, particularly monocytes and neutrophils, which are also actively involved in inflammatory processes [28].

 It is important to note that the glial and inflammatory response after perinatal brain damage differs from the mature brain [25] due to key ongoing postnatal developmental processes. Importantly, neuronal dendritic arborization, establishment of synaptic contacts, axonal growth, myelination, and glial differentiation take place during the first two-three postnatal weeks in rodents [29]. At the molecular level, several studies have described a distinctive expression of growth factors [30], adhesion molecules [31], inhibitors of axonal growth [32], and cytokines [33–35], determining the neonatal brain's particular response to injury, showing increased susceptibility to excitotoxicity [11, 36, 37] and to proinflammatory molecules [38, 39]. In this regard, it becomes evident that descriptions of the glial and inflammatory cell changes in adult injury models cannot be extrapolated to animal models of perinatal brain damage.

 In the present study we have used the experimental model of H/I-induced neonatal injury initially described by Vannucci and coworkers [17, 40] for the rat, and adapted to the mouse in several laboratories [14, 18, 41] with the advent and increased usage of transgenic and knock-out mice. As most studies describing detailed neuropathological and glial and inflammatory cellular changes after neonatal H/I have used the rat model, the goal of our study was to provide a neuropathological followup of tissue damage and detailed morphological and quantitative analysis of astroglial, microglial and leukocytic response following H/I to the postnatal day 7 mice at nine different survival times ranging from 3 hours after hypoxia to 100 days, focusing both on the ipsilateral and the contralateral hemispheric changes. This short- and long-term temporal description aims to help in the future design of novel experimental approaches towards the development of neuroprotective strategies.

## 2. Materials and Methods

### 2.1. Animals

Ninety-nine C57BL6 mice (from twenty litters bred in Harlan Labs, France) of different postnatal ages were used in this study. Experimental animal work was conducted according to Spanish regulations following European Union directives. Animals were housed under controlled temperature (22°C ± 2°C), with a 12 hour light cycle period and pelleted food (Global diet 2014) and water *ad libitum*. The dams and pups were kept on enriched environment. Experimental procedures were approved by the ethical commission of Autonomous University of Barcelona (CEEAH protocol no. 811). All efforts were made to minimize the number of animals and animal suffering in every step.

### 2.2. Hypoxia/Ischemia

Hypoxic/ischemic (H/I) brain damage was induced in postnatal day 7 (P7) C57/BL6 mice by permanent left carotid occlusion and exposure to hypoxia as previously described [42]. Briefly, a midline ventral skin incision was made under isoflurane anesthesia (4.5% v/v for induction and 2.5% v/v for maintenance, and 0.6 L/min of O_2_); the left carotid artery was exposed and sutured with a 8/0 silk surgical suture. After surgery, pups were returned to their dam for at least 1.5 hours to recover. Later, litters were placed for 55 minutes in a hypoxic chamber containing 8% of oxygen balanced with nitrogen, with controlled humidity and temperature maintained at 37°C. Pups were then returned to their dam until sacrifice. The mean index of postnatal mouse mortality due to surgery or hypoxia was 19.31%, with 18.46% for males and 20.00% for females, showing no statistical differences between genders. As 18 animals died during surgical procedure or hypoxia, only 81 animals were analyzed in this study.

### 2.3. Groups and Sample Processing

Intact control mice were sacrificed at P7, P10, P14, P21, and adult. Lesioned pups were sacrificed at 3, 12, 24, 48, and 72 hours, and at 7, 14, 30, and 100 days after hypoxia. All survival times included pups from at least 3 different litters. Animals were grouped as follows for comparison and analysis with controls: Group I—P7, 3 hrs, 12 hrs, 24 hrs; Group II—P10, 48 hrs, 72 hrs; Group III—P14, 7 days; Group IV—P21 and/or adult, 14 days, 30 days, and 100 days. For histological and immunohisto-chemical analysis, mice were i.p. anaesthetized (ketamine and xylazine 80/10 mg/Kg) and perfused intracardially using 4% paraformaldehyde in phosphate buffer (PB, pH 7.4). Subsequently, brains were removed, postfixed for 4 hours in the same fixative, cryoprotected in 30% sucrose, frozen with dry CO_2_, and finally stored at −80°C until use. Brains were serially cut in a cryostat (Leica CM3050 S) in 30 *μ*m thick sections and stored in −20°C mounted on Flex IHC slides (Dako).

### 2.4. Nissl Staining: Evaluation of Injury Score

To determine the injury score, slides were processed for Nissl staining. One series of parallel sections from each animal (6–10 mice/survival time) was air dried at room temperature for an hour, rinsed and incubated with Nissl solution (0.1% toluidine blue in walpole buffer 0,2 M and pH 4,5) at room temperature for 3 minutes and washed with distilled water. Sections were dehydrated, cleared in xylene, and coverslipped with DPX. The degree of tissue damage was calculated following the injury score detailed on Table 1 (for 3 to 72 hrs) and Table 2 (for 7 to 100 days).

### 2.5. Immunohistochemistry

Three animals from each control age group and four representative animals from each postlesion survival time (injury scores = mean ± 2 S.D.) were processed for the immunohistochemical demonstration of astrocytes (by glial fibrillary acidic protein, GFAP labeling), microglia/macrophages (by Iba-1 labeling), neutrophils (by Ly-6B.2 labeling), and T-cells (by CD3 labeling). Single immunohistochemistry was initiated by blocking the endogenous peroxidase (2% H_2_O_2_ in 70% methanol for 10 min) and incubation of sections mounted on slides for 1 h in blocking buffer (BB) containing 10% fetal calf serum and 3% bovine serum albumin in tris-buffered saline (TBS, pH 7.4) with 1% Triton X-100 (TBST) at room temperature (RT). Slides were then incubated overnight at 4°C and 1 h at RT with one of the following primary antibodies diluted in BB: hamster monoclonal anti-CD3 (AbD Serotec no. MCA2690, dilution 1 : 250), rabbit polyclonal anti-GFAP (DAKO no. Z0334, dilution 1 : 1500), rabbit polyclonal anti-Iba-1 (Wako no. 019-19741, dilution 1 : 3000), and rat monoclonal Ly-6B.2 (AbD Serotec no. MCA771G, dilution 1 : 500). Later, sections were washed with TBST and incubated at RT for 1 h with respective biotinylated secondary antibodies: anti hamster (Vector Labs no. BA9100, dilution 1 : 500), anti-rabbit (Vector Labs no. BA1000, dilution 1 : 500), and anti-rat (Vector Labs no. BA4001, dilution 1 : 500), followed by washes with TBST and incubation for 1 h with streptavidin–peroxidase (Vector Laboratories no. SA-5004, dilution 1 : 500). The peroxidase reaction was visualized by incubating the sections in 3,3-diaminobenzidine and hydrogen peroxide using the DAB kit (SK-4100; Vector Laboratories, USA) for GFAP, Iba-1 and Ly-6B.2. For CD3, slides were treated by the glucose oxidase-DAB-nickel method [43], and the reaction was terminated by washing with 0.1 M acetate buffer (pH 6.0). Finally, sections were dehydrated and coverslipped in DPX. Sections were analyzed and photographed with a DXM 1200F Nikon digital camera joined to a Nikon Eclipse 80i microscope, and plates were arranged using Adobe Photoshop CS.

### 2.6. Quantitative Analysis of Immunohistochemical Labelling

**Figure 1:**
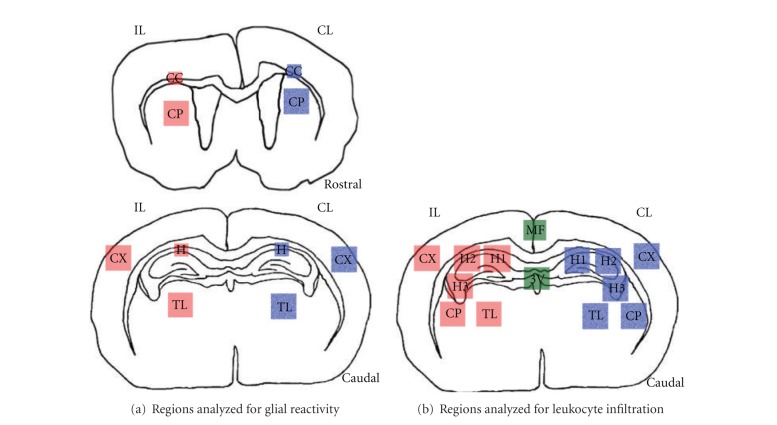
Drawings modified in Adobe Photoshop CS showing brain areas analyzed for quantification of different antibodies used in the study. (a) Regions analyzed for quantification of glial reactivity in rostral and caudal side of the brain (see Section 2 for details). (b) Regions analyzed for quantification of leukocyte infiltration. The regions in red are from the ipsilateral (IL) side and the regions in blue are from the contralateral (CL) side while the medial regions are shown in green. CC: corpus callosum; CP: caudate-putamen; CX: cortex; H: hippocampus; MF: medial fissure; TL: thalamus; 3V: third ventricle.

ImageJ software (National Institute of Health) was used for quantitative analysis of immunoreacted sections. At least 4 animals/lesioned groups were analyzed. Images from 5 sections/animal were taken, representing the following regions: corpus callosum (CC), caudate putamen (CP), hippocampus (H), neocortex (N), and thalamus (T) (Figure 1). Micrographs were captured using the 40x objective (for the CC and the hippocampus at 72 hours after hypoxia) or the 20x objective (rest of areas and survival times). In group I, II and III sections were 240 *μ*m apart, and bregma levels (BLs) analyzed included (approx.): *Anterior*—BL1, 0.26 mm & BL2, 0.02 mm; *Posterior*—BL3, −1.82 mm; BL4, −2.06 mm; BL5, −2.30 mm. In Group IV, sections were 300 *μ*m apart, and BL analyzed included: *Anterior*—BL1, 0.32 mm & BL2, 0.02 mm; *Posterior*—BL3, −1.82 mm; BL4, −2.12 mm; BL5, −2.42 mm. Image analysis was used to obtain the area occupied by glial cells, using a modification from a previously described method [44]. Initially, in each section, the mean intensity of grey (immunoreactive labeling) in the contralateral region was measured. Subsequently, by using the mean intensity of grey as the threshold value, we measured in both hemispheres the percentage of the total area occupied by immunoreactive staining showing an intensity of grey above the threshold (i.e., representing reactive cells). All samples for demonstration of atrocytes and microglia were done simultaneously in order to reduce variability on DAB intensity. Data of both ipsilateral and contralateral hemispheres are shown as mean values ± S.E.M.

### 2.7. Neutrophil Cell Counting

Neutrophils were counted using ImageJ software (National Institutes of Health). The regions analysed are shown in Figure 1(b) and included the hippocampus (H1, H2, H3), neocortex (N), caudate-putamen (CP), medial third ventricle (M3V), lateral third ventricle (L3V) median fissure (MF), and thalamus (T) in at least 4 representative animals of each lesioned group and 3 animals/control group, with 3 sections/animal, was analysed. In groups I, II and III sections were 240 *μ*m apart, and counted bregma levels (BLs) included: BL1, −1.82 mm; BL2, −2.06 mm; BL3, −2.30 mm. In Group IV, sections were 300 *μ*m apart, and counted BL included: BL1, −1.82 mm; BL2, −2.12 mm; BL3, −2.42 mm. All data was corrected by Abercrombie correction method [45], with an average of length (*t*) = 0,848. Data is presented as mean number of cells/mm^2^.

### 2.8. Statistical Analysis

All experiments were performed so as to reduce variations, and data are presented as mean ± S.E.M. The data was considered significant at *P*-value <0.05. Two-way ANOVA followed by Bonferroni *posthoc* analysis, along with *t*-test, was used to determine statistical significance as required (Graphpad, Prism 3).

## 3. Results

### 3.1. Tissue Damage and Injury Score

**Figure 2:**
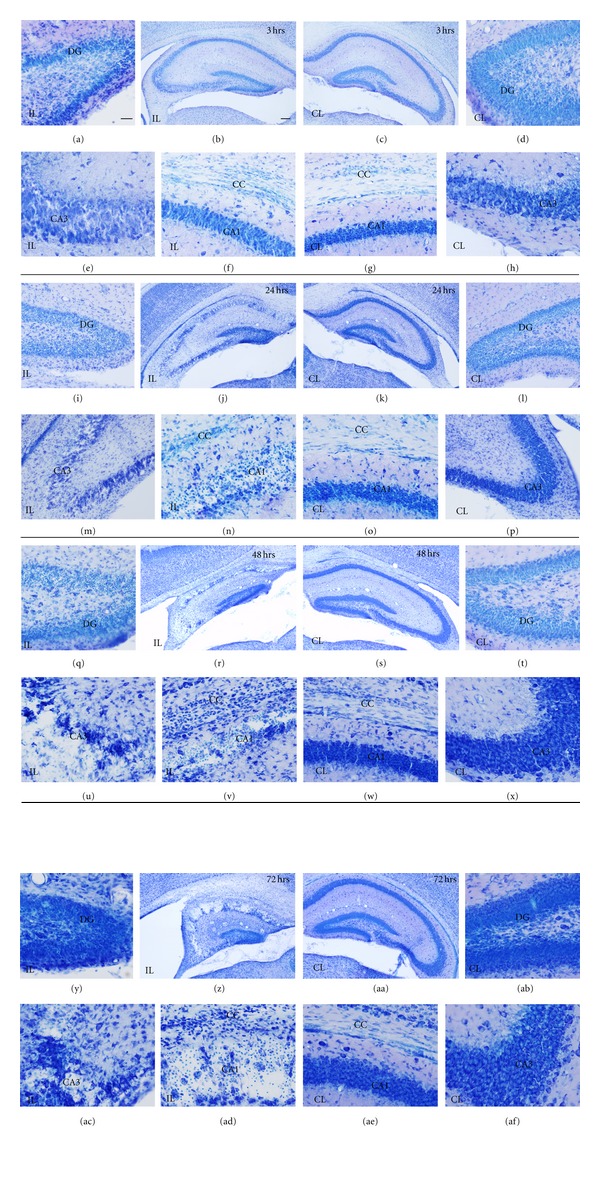
Nissl staining showing hypoxia/ischemia (H/I) effects on the hippocampus and corpus callosum of the contralateral (CL) (right side of the panel), and ipsilateral hemisphere (IL) (left side of the panel), from 3 to 72 hours (hrs) after hypoxia. At 3 hrs (a–h), layer disruption is seen in ipsilateral CA3 (e) and increased cellularity in the ipsilateral corpus callosum (f). At 24 hrs (i–p) neuronal degeneration is widespread in hippocampus (i, j, m, and n). At 48 hrs (q–x) and 72 hrs (y–af), hippocampal atrophy (compare r to s, z to aa) and massive neuronal loss is seen in CA1 and CA3 (r, u, and v for 48 hrs, z, ac and ad for 72 hrs) although the DG is also disorganized (compare q to t, y to ab). Increased cellularity in the corpus callosum is also seen (v and ad). Scale bars (low magnifications: b, c, j, k, r, s, z, aa) = 100 *μ*m; scale bar in all other micrographs = 25 *μ*m. CA1: cornu ammonis 1; CA3: cornu ammonis 3; CC: corpus callosum; DG: Dentate gyrus.

**Figure 3:**
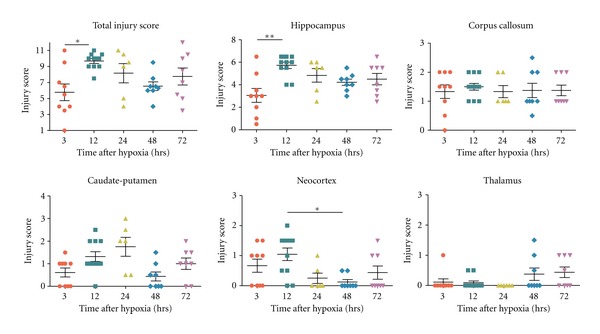
Graphs showing the changes in the total injury score along with the injury score in different regions analyzed after 3 to 72 hours (hrs) after hypoxia in Nissl-stained coronal sections. Kruskal Wallis test was done followed by Dunn's multiple comparison test. *P < 0.05, **P < 0.01 was considered significant.

**Figure 4:**
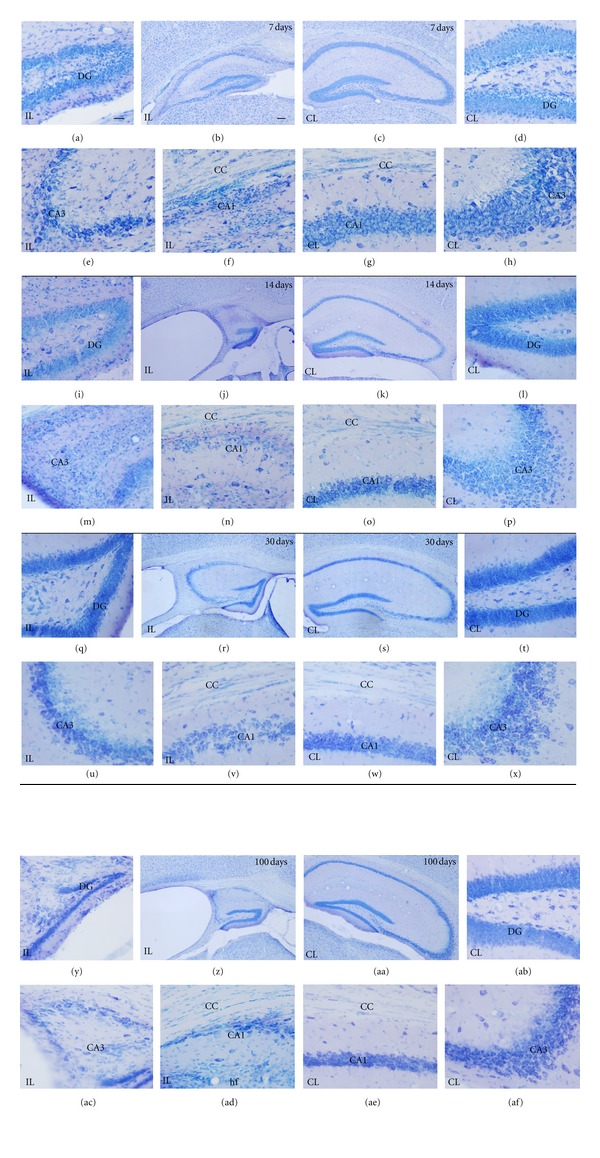
Nissl staining showing H/I effects on the hippocampus and corpus callosum of the contralateral (CL) (right side of the panel), and ipsilateral hemisphere (IL) (left side of the panel), from 7 to 100 days (d) after hypoxia. At 7 d (a–h), overall hippocampal atrophy (b), layer disruption in ipsilateral CA3 (e), and increased cellularity in the ipsilateral corpus callosum (f) are seen. At 14 d (i–p) neuronal degeneration is widespread in hippocampus (i, j, m, and n). At 30 d (q–x) and 100 d (y–af), hippocampal atrophy (compare r to s, z to aa) and massive neuronal loss is seen in CA1 while CA3 is almost disorganized (r, u, and v for 30 d, z, ac, and ad for 100 d), along with the disorganized DG (compare q to t, y to ab). Increased cellularity in the corpus callosum is also visible (v and ad). Scale bars (low magnifications: b, c, j, k, r, s, z, aa) = 100 *μ*m; scale bar in all other micrographs = 25 *μ*m. CA1: cornu ammonis 1; CA3: cornu ammonis 3; CC: corpus callosum; DG: Dentate gyrus.

**Figure 5:**
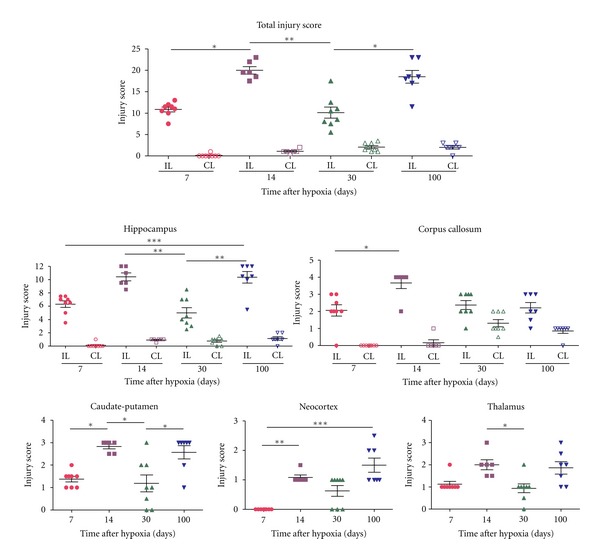
Graphs showing the changes in the total injury score along with the injury score in different regions analyzed after 7 to 100 days after hypoxia following Nissl staining. Kruskal Wallis test was done followed by Dunn's multiple comparison test. *P < 0.05, **P < 0.01, ***P < 0.001 is considered significant.

Analysis of toluidine blue-stained sections (Figures 2 and 4) was used to evaluate the extent of brain damage in both hemispheres at 3, 12, 24, 48, and 72 hours and at 7, 14, 30, and 100 days after hypoxia. In general, microscopic evaluation showed mild changes in the contralateral hemisphere [mainly in hippocampus (HP) and corpus callosum (CC)], and extensive tissue damage and neuronal loss in the ipsilateral HP and CC at all survival times analyzed, although the caudate putamen (CP) was also usually affected. Damage in the cortex (CX) and the thalamus (TL) was not always seen and showed the highest variability. In order to better characterize lesion progression, a semiquantitative injury score was calculated for each region and animal (Tables 1 and 2, Figures 3 and 5). From 3 to 72 hours after hypoxia, damage was characterized by neurodegeneration and increased cellularity due to gliosis, and the description of the injury score rating is depicted in Table 1. At 7 days after hypoxia, damage was mainly characterized by atrophy of gray and white matter areas, and therefore a different injury score rating was defined, which is depicted in Table 2.

#### 3.1.1. Tissue Damage in the Contralateral Hypoxic Hemisphere

From 3 hours to 7 days after hypoxia, no apparent tissue damage or ventricle swelling in the contralateral hemisphere was observed using the Nissl staining (Figures 2 and 4; right side of the panel). Interestingly, at 14 days after hypoxia, scattered patches of neurodegeneration with a mild reduction in cellular density when compared to intact age-matched control brains were observed in the CA field of the HP (Figure 4), showing a mean injury score in the contralateral HP of 0.92 ± 0.2 (Table 2, Figure 5). In addition, the contralateral CC was also damaged in the 30- and 100-day survival groups, showing approximate 40% of atrophy (mean CC atrophy scores of 1.31 ± 0.59 and 0.86 ± 0.38, resp.) (Table 2, Figure 5), accompanied by evident ventricle swelling (Figure 4). No apparent changes in the contralateral dentate gyrus (DG), caudate-putamen, neocortical layers and thalamus were seen.

#### 3.1.2. Tissue Damage in the Ipsilateral Hypoxic/Ischemic Hemisphere

**H/I Injury in Hippocampus**

As early as 3 hours after hypoxia, hippocampal tissue disruption with disorganization of CA cytoarchitecture and the presence of patches of neurodegeneration CA pyramidal neurons was observed in the ipsilateral hemisphere (Figures 2(b), 2(e) and 2(f)), but showing a high degree of variability between animals (Figure 3). From 12 to 72 hours after hypoxia, the hippocampal CA field was visibly damaged in all animals, displaying a degenerating pyramidal cell layer with massive neuronal cell loss in CA1 and CA3 (Figures 2(i)–2(af), left panel), showing a mean injury score of CA field of 3.27 ± 0.74 between 12 and 72 hours after hypoxia (Table 1, Figures 2 and 3). In addition, at 12 hours, the dentate gyrus (DG) also showed neuronal injury and layer disruption, which was most evident at the 12- and 24-hours survival times (Figure 2(i)). At 7 days after hypoxia, massive atrophy of the hippocampus was observed, showing mean total hippocampal injury scores ranging from  5 ± 2.2  to 10.42 ± 1.46 (out of 12, Table 2), where the 30-day survival group showed the lowest score (Figures 4 and 5). Hippocampal damage induced approximately a 10–40% of remaining CA pyramidal neurons, but less than 50% reduction in DG neuronal density (Figures 4 and 5). Interestingly, only in the 33% of the animals, the ipsilateral hippocampus was observed 100 days after hypoxia.

**H/I Injury in Corpus Callosum**

From 3 hours post-hypoxia, the ipsilateral corpus callosum showed increased cellularity (Figure 2(f)) and the presence of scattered apoptotic cells (data not shown). The density of cells in the ipsilateral corpus callosum was notably increased at 48 and 72 hours post-hypoxia (Figures 2(v), 2(ad) and 3), when ventricle swelling started to become evident (Figures 2(r) and 2(z)). At 7 and 14 days post-hypoxia, increased cellularity was still observed (Figures 4(f) and 4(n)), but this was minimum from 30 days (Figure 4(v)). Important atrophy of the white matter accompanied by ventricle swelling was seen in all animals at 7 days after hypoxia, but it was more remarkable at 14 days after hypoxia, showing mean corpus callosum atrophy score (14–100 days) of 2.33 ± 0.84, which represented an approximate 50% tissue loss (Table 2, Figure 5).

**H/I Injury in Caudate-Putamen, Neocortex, and Thalamus**

At 3 hours after hypoxia, we observed increased cellularity and disorganization of white and gray matter areas, mainly in the dorsal part of caudate-putamen (Figure 3), showing a mean injury score (3–72 hours) of 1.02 ± 0.81 corresponding to less than 40% of striatal area damaged (Table 1, Figure 3), but showing important variability between animals (Figure 3). At 7 to 100 days after hypoxia, there was apparent caudate-putamen atrophy (Figures 3 and 5).

The neocortex and the thalamus showed mild changes, that were only apparent in a minority of animals at all times analyzed, giving very variable results (Figures 3 and 5). Neocortical damage, when present, was characterized by scattered radial columns of neurodegeneration and tissue damage, mainly until 12 hours after hypoxia. At 7 days after hypoxia mild atrophy was seen in some cases (Figure 5). Cellular damage in the thalamus was even less frequent but could be observed in some animals, affecting the rostral thalamic nuclei (Figure 3). However, probably as a consequence of ventricle swelling, different grades of thalamic atrophy were seen in most animals at 7 days (Table 2, Figure 5).

### 3.2. Astroglial Response

Astrocytes were analyzed by GFAP immunostaining and studied in control intact brains from P7, P10, P14, P21 and adult mice, and in the contralateral and ipsilateral hemisphere of hypoxic/ischemic brains from 3 hours to 100 days after hypoxia.

#### 3.2.1. GFAP*+* Cells in the Control Postnatal Brain

**Figure 6:**
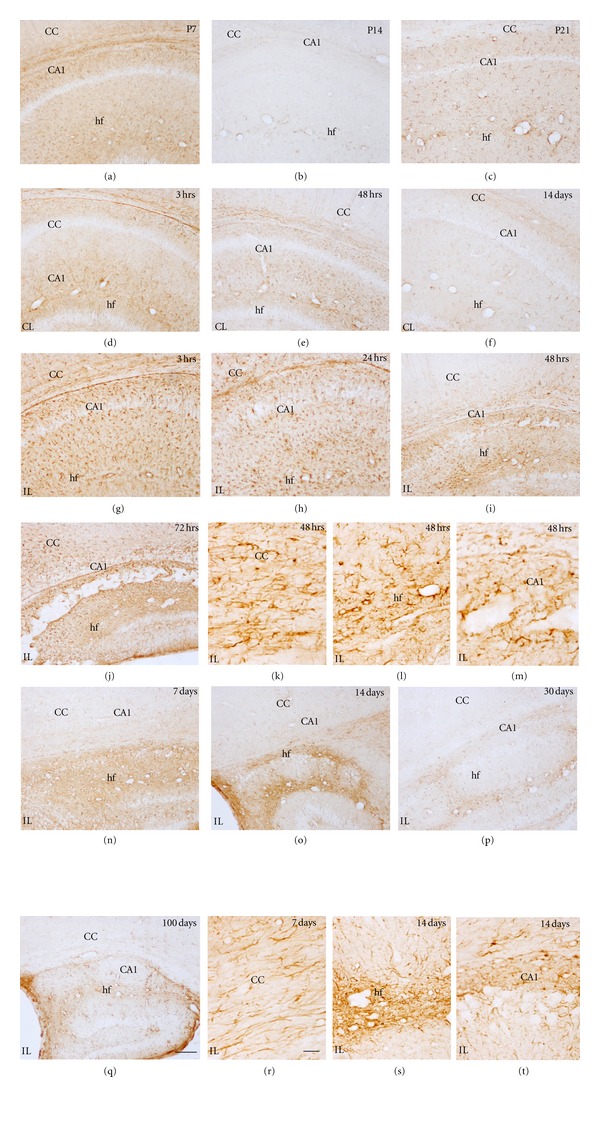
GFAP immunostaining showing age-matched controls and effects of H/I on the hippocampus and corpus callosum. Developmental changes in astrocytes are observed in control animals (a–c), showing a progressive change from more activated astrocytes (a) to resting astrocytes at P14 (b) and P21 (c). Activated astrocytes increase in the contralateral (CL) (d–f), and ipsilateral hemisphere (IL), from 3 hours (hrs) to 100 days after hypoxia (g–t). At 3 hrs (g), astrocyte activation can be seen in the hippocampal fissure, CA and corpus callosum. At 48 hrs (i, m) and 72 hrs, (j) CA layer degeneration is observed. From 7 days (n) to 100 days (t), there is a decrease of the astrocytes activation in the corpus callosum, but in the hippocampus the reduction in astrogliosis starts at 30 days after hypoxia (p). Scale bars (low magnifications: a–j, n–p and t) = 100 *μ*m; scale bar in all other micrographs = 20 *μ*m. CA1: cornu ammonis 1; CC: corpus callosum; hf: hippocampal fissure.

**Figure 7:**
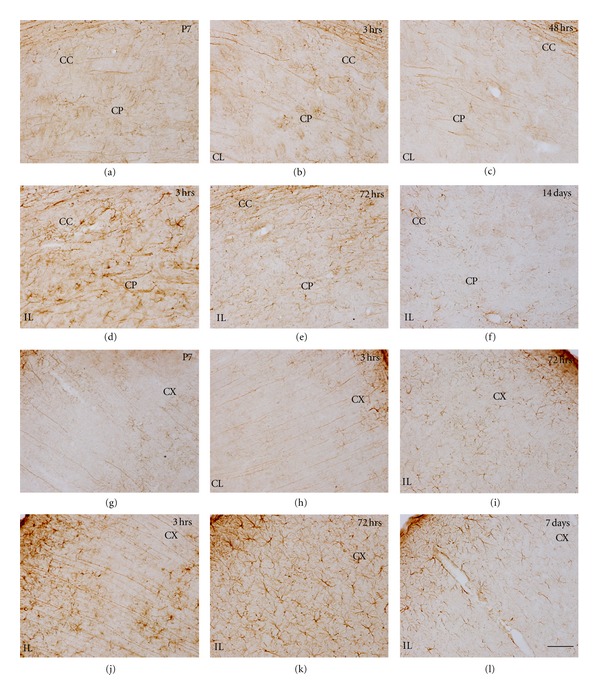
GFAP staining showing effects of H/I on the caudate-putamen (a–f) and cortex (g–l) of the contralateral (CL) (b, c and h, i) and ipsilateral hemisphere (IL) from 3 hours (hrs) to 14 days after hypoxia (d–f and j–l). Reactive astrocytes are seen in ipsilateral side from 3–72 hrs (d, e) as compared to the P7 control (a) or the contralateral side (b, c). At 14 days after hypoxia the astrocytes reactivity decreases (f). The cortex at 3 hrs (j) shows increase in reactive astrocytes and radial glia-like structures (only at this age) as compared to P7 control (g) and contralateral side (h) with a maximum reactivity at 72 hrs (k) when compared to the respective contralateral side (i); and a decrease in astroglial reactivity is seen at 7 days after hypoxia (l). Scale bar = 100 *μ*m. CP: caudate-putamen; CC: corpus callosum; cx: neocortex.

The distribution and immunostaining intensity of GFAP+ cells changed during postnatal development (Figures 6(a)–6(c)), showing increased GFAP levels at earlier ages, as has been previously reported [46–49]. Briefly, in addition to the GFAP+ radial glial processes still observed at P7 (Figure 7(g)), at the P7–P10 age range, the most intense GFAP+ astroglial cells were found in cortical layer I, the hippocampal fissure (Figure 6(a)) and white matter areas including the corpus callosum (Figure 6(a)), and the fimbria. At P14, GFAP immunoreactivity was generally decreased but it was maintained in cortical layer I, the hippocampal fissure and white matter tracts (Figure 6(b)). By P21 in the adult pattern of GFAP+ cell distribution was established, showing the strongest immunoreactivity in the astroglial endfeet surrounding blood vessels (as in the hippocampal fissure, Figure 6(c)) and in the white matter.

#### 3.2.2. Astroglial Changes in the Contralateral Hypoxic Hemisphere

An astroglial response in the contralateral hemisphere was generally observed, mainly from 3 to 72 hours after hypoxia, and being importantly decreased by 7 days and longer survival times. Increase in GFAP immunoreactivity due to astrogliosis was mainly seen in the hippocampal region (mainly in the hippocampal fissure and the fimbria) and in the cingulum region of the corpus callosum (Figures 6(d)–6(f) compared to age-matched controls in 6(a)–6(c)). Astroglial changes in the contralateral hippocampus were maximal at 24–48 hours after hypoxia (Figure 6(e)). In addition, mild changes were also noted in the neocortex (Figures 7(h) and 7(i)), but no apparent changes were observed in the contralateral caudate-putamen (Figures 7(b) and 7(c)) and thalamus. At 7 days after hypoxia, contralateral hemispheres showed no changes in GFAP+ cell distribution when compared to age-matched controls. In this sense, it is important to note that the contralateral hippocampal and corpus callosum atrophy observed from 14 days post-hypoxia (Figure 5) was not accompanied by noticeable astroglial changes in these areas at late survival times.

#### 3.2.3. Astroglial Changes in the Ipsilateral Hypoxic/Ischemic Hemisphere

**Figure 8:**
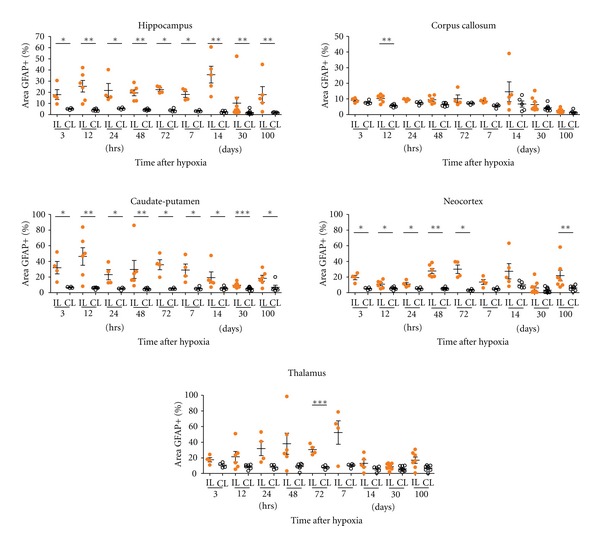
GFAP+ area on the hippocampus, corpus callosum, caudate-putamen, neocortex, and thalamus is evaluated from 3 hrs to 100 days after hypoxia in the ipsilateral (IL) and contralateral (CL) hemispheres. Astrogliosis is shown as the percentage of the GFAP+ area (see Section 2 for details). The hippocampus and caudate-putamen are the most affected regions and significant differences between IL and CL hemispheres are found at all time points. The astroglial reactivity in corpus callosum is observed at 12 hrs after hypoxia between IL and CL. Higher astrogliosis is observed in the ipsilateral neocortex at almost all times analyzed compared to CL side. A significant increase in astroglial activation after 72 hrs is seen in the IL thalamus. Significant differences between IL and CL hemisphere are shown using unpaired *t* tests, with Welch's correction if suitable (*P < 0.05, **P < 0.01, ***P < 0.001). Individual data and mean ± S.E.M, are represented to show the dispersion in each group.

Increased GFAP immunostaining and changes in astroglial distribution and astrogliosis were seen in the ipsilaterally damaged hemisphere from 3 hours to the last survival time analyzed (Figures 6–8). The most intense astroglial response was found in the damaged hippocampus although the corpus callosum, the caudate-putamen, the neocortex and the thalamus also showed noticeable astroglial reactivity.

#### 3.2.4. Hippocampus

At 3 hours after hypoxia, the ipsilateral hemisphere already showed an increase in astroglial GFAP labeling as well as astrogliosis when compared to the contralateral side (Figures 6(d) and 6(g)). At this survival time, and at 12 hours after hypoxia, reactive astrocytes mainly covered the hippocampal fissure, and the molecular and polymorphic layers of the CA field, but no reactive astrocytes were seen within CA pyramidal cell layer or in the DG. At these early survival times, the area occupied by reactive astrocytes was significantly increased in the IL side (Figure 8). At 24 hours, but mainly at 48–72 hours after hypoxia, astroglial processes started to cover the degenerating CA1 and CA3 pyramidal layers and reactive astrocytes concentrated in the hippocampal fissure, the molecular layer and the polymorphic layer of CA1, adjacent to the white matter (Figures 6(h)–6(m)). Astroglial cell response was at this time also evident, to a lower extent, in the DG, mainly in the hilus (Figure 6(j)). As depicted in Figure 8, the percentage of GFAP+ area in the hippocampus was high and significant in IL hippocampus at all survival times. At 7 days after hypoxia, an intense glial scar formed in the degenerated pyramidal layer, around the blood vessels in the hippocampal fissure and in the hippocampal limits (Figure 6(n)). At 14 days after hypoxia, astroglial response in the DG was noticeably decreased although increased GFAP+ cells were often seen in the hilus (Figures 6(o), 6(s)–6(t)). The glial scar was maintained until 100 days after hypoxia (Figures 6(p) and 6(q)).

#### 3.2.5. Corpus Callosum

An increase in GFAP immunostaining and cell density when compared to the contralateral side was already seen at 3 hours after hypoxia (Figure 6(d) and 6(g)), however maximum response was observed at 24–72 hours after hypoxia (Figures 6(h)–6(k)), when reactive astrocytes presented a marked increase in GFAP intensity, showing hypertrophy and increased process thickness. By 7 days, astrogliois clearly diminished (Figures 6(n) and 6(q)), and at 14 days after hypoxia, GFAP immunostaining was strongly decreased and was indistinguishable from controls (Figures 6(o)–6(r)). It should be noted that no striking changes were observed in the quantification of the astroglial response when compared to the contralateral side (Figure 8).

#### 3.2.6. Caudate-Putamen, Neocortex, and Thalamus

An increase in astroglial GFAP immunoreactivity was noted in the caudate-putamen at 3 hours after hypoxia (Figure 7(d)) although no changes in astroglial distribution were seen until later. From 24 hours, astroglial response was mildly increased until 72 hours, when maximum GFAP labeling was reported (Figure 7(e)). Astroglial GFAP expression was close to control values by 14 days after hypoxia (Figure 7(f)), although glial scarring in the caudate-putamen remained in some animals at longer survival times, showing variability (Figure 8). Notably, the area occupied by reactive astroglial cells in the ipsilateral caudate-putamen was above contralateral values at all survival times analyzed even though variability was found in some time points (Figure 8).

 In the neocortex, increased GFAP expression and mild astrogliosis were first observed in layers V-VI at 3–12 hours after hypoxia (Figure 7(j)), and it spread to upper layers from 24 to 72 hours (Figure 7(k)), showing significant increases in astroglial response area (Figure 8). At longer survival times, astrocytic response was clearly diminished (Figures 7(l) and 8) and was practically absent by 14 days after hypoxia. In the thalamus, changes in astrocytes were not observed until 24 hours after hypoxia, showing strong variability between animals (Figure 8). Astroglial response was characterized by patches of reactive astrocytes mainly in the rostral thalamus and only until 7 days after lesion, when glial scarring was noticed. At longer survival times, it was clearly diminished.

### 3.3. Microglia/Macrophage Response

#### 3.3.1. Iba1+ Microglia/Macrophages Cells in the Control Postnatal Brain

**Figure 9:**
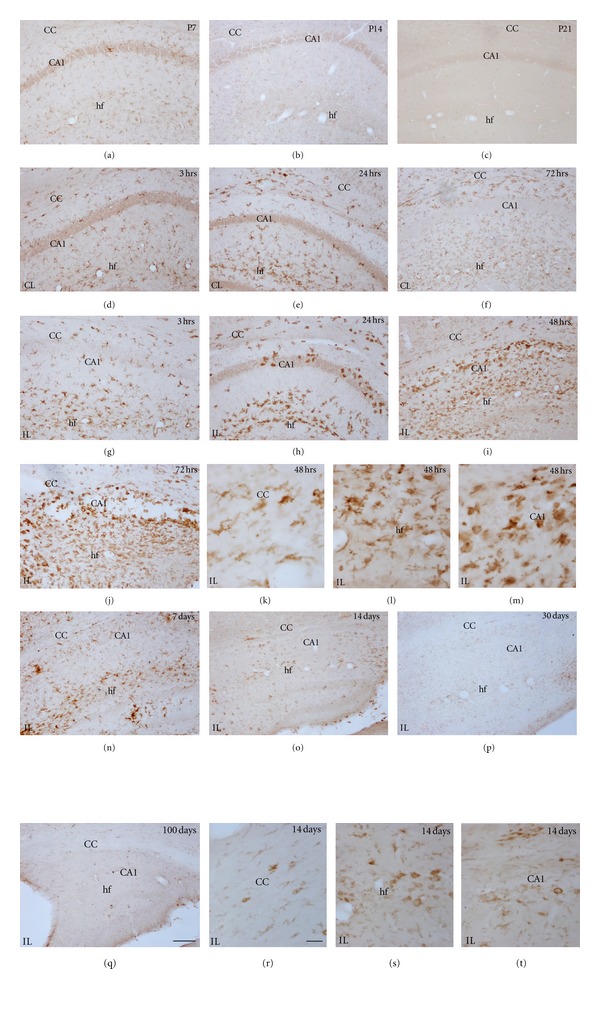
Iba-1 immunostaining showing age-matched controls and H/I effects on the hippocampus and corpus callosum. Developmental changes in microglia are observed in control animals (a–c), showing a progressive change from mainly primitive ramified and amoeboid cells (a) to a resting morphology at P14 (b) and P21 (c). Activated microglia increases in the contralateral (CL) (d) and ipsilateral (IL) hemisphere at 3 hrs (g). At the CL side, Iba-1 shows the maximum labeling at 24 hrs (e) in the hippocampal fissure, returning to control level at 72 hrs (f). In the IL side, higher proportion of amoeboid cells is seen from 24 hrs (h) to 72 hrs (j). At 7 days after hypoxia (n), a reduction on the level of microglia morphologically activated is observed, returning to the basal level at 14 days (o). Resting morphology is observed at 30 (p) and 100 days (q). Detailed morphology of Iba-1+ cells in the cc (k), hf (l), and CA1 (m) observed after 48 hrs and 14 days after H/I (r, s and t, resp.) are shown. Scale bars: in low magnifications: (a) to (j) and (n) to (q) = 100 *μ*m; scale bar in (k, l, m r, s, and t) = 20 *μ*m. CA1: cornu ammonis 1; hf: hippocampal fissure; CC: corpus callosum.

**Figure 10:**
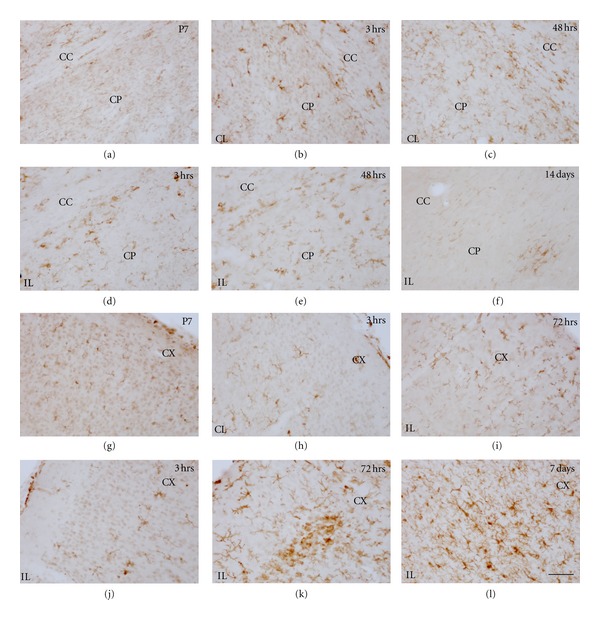
Iba-1 immunostaining in control animals and H/I effects on the caudate-putamen (CP) (a–f) and neocortex (CX) (g–l). Control brain P7 shows basal expression of microglial Iba-1 in the CP (a) and CX (g). In the CP, activated microglia increase in the contralateral (CL) hemisphere at 3 hrs (b) up to 48 hrs (c). Higher activation is observed in the ipsilateral (IL) hemisphere at 3 hrs (d), showing a maximum at 48 hrs (e), and slowly returns to resting morphology with some patches of activated microglia after 14 days (f). Similar pattern is observed in the neocortex, CL hemisphere shows differential expression with respect to control animals from 3 hrs (h) to 72 hrs (i). Higher activation is observed in the ipsilateral (IL) hemisphere at 3 hrs (j), a clear amoeboid patch pattern is observed in the neocortex after 72 hrs (k), which slowly returns to resting morphology with some patches of primitive ramified microglia after 7 days (l). Scale bars: 50 *μ*m.

Intense microglial Iba-1 staining was observed at P7 and gradually decreased until adulthood. In postnatal animals, primitive ramified microglial cells were mainly found in the gray and white matter (Figures 9(a), 10(a) and 10(g)) [50] although some amoeboid microglial cells were seen in the cingulum of the corpus callosum, as previously reported [51]. In the hippocampus, the number of microglial cells gradually increased from medial to lateral regions. In addition, round-shaped Iba-1+ macrophages were observed in the pia, very prominently in the medial fissure and in the ventricle linings, as has already been reported [52]. At P10, microglial cells were slightly more ramified, and an increase in cell density was noted, specifically in the corpus callosum, where microglial cells showed a parallel orientation to axon fibers. By P14, microglial cells showed decreased Iba-1 immunostaining (Figure 9(b)) and ramified resting morphology as described for the adult brain [50]. At this age, Iba-1+ macrophages were strongly diminished in the meninges and ventricles. By 21 days after birth, only highly ramified resting microglial cells were observed in the brain parenchyma, showing very low Iba-1 staining (Figure 9(c)).

#### 3.3.2. Microglia/Macrophage Changes in the Contralateral Hypoxic Hemisphere

Microglial activation was generally observed in several areas of the contralateral hemisphere from 3 to 48 hours after hypoxia (Figures 9(d) and 9(e)). Increased expression of Iba-1 and changes in microglial cell morphology towards reactive ramified cells mainly, but also amoeboid cells to a lower extent, were seen in most areas analyzed, but mainly in the hippocampus (very prominently in the hippocampal fissure, Figures 9(d) and 9(e)) and the corpus callosum (Figures 9(d)–9(f)) and other white matter tracts like the anterior commissural and external capsule, where microglial response was seen until 48–72 hours after hypoxia. After 14 days fter hypoxia, only in the hippocampal fissure and corpus callosum of some animals, mild-activated microglia was observed. In the caudate-putamen (Figures 10(b) and 10(c)), neocortex (Figures 10(h) and 10(i)) and thalamus (data not shown), activated ramified microglial cells were seen mainly until 48 hours after hypoxia.

#### 3.3.3. Microglia/Macrophage Changes in the Ipsilateral Hypoxic/Ischemic Hemisphere

**Hippocampus**

**Figure 11:**
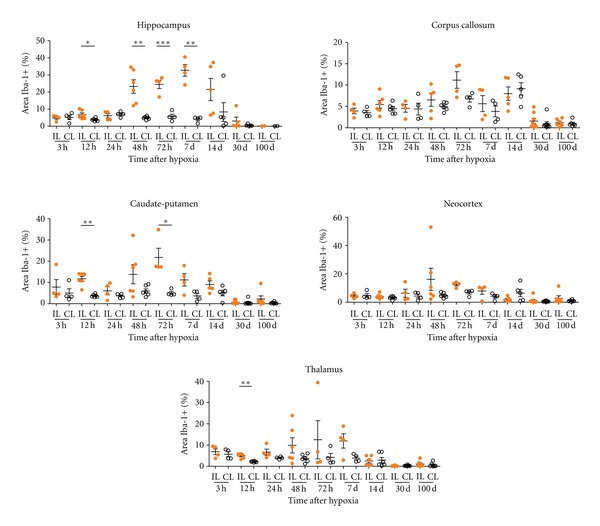
Iba-1+ area on the hippocampus, corpus callosum, caudate-putamen, neocortex, and thalamus is evaluated from 3 hrs to 100 days after hypoxia in the ipsilateral (IL) and contralateral (CL) hemispheres. Reactive microglia is shown as the percentage of the Iba-1+ area (see Section 2 for details). The hippocampus is the most affected region by H/I at 12, 48, 72 hrs and 7 days, as can be observed between IL and CL hemisphere. Significant differences on Iba-1+ area are observed at 12 and 72 hrs after hypoxia in the caudate-putamen and after 12 hrs in the thalamus. Significant differences between IL and CL hemisphere are shown using unpaired *t* tests, with Welch's correction if suitable (*P < 0.05, **P < 0.01, ***P < 0.001). Individual data and mean ± S.E.M, are represented to show the dispersion in each group.

At 3 hours after hypoxia, microglial response in the ipsilateral hippocampus closely resembled that seen in the contralateral side; however, reactive microglial cells tended to accumulate surrounding the blood vessels in the hippocampal fissure only in the ipsilateral hippocampus (Figure 9, compare 9(d) and 9(g)). By 12 hours, reactive microglial cells changed to pseudopodic/ameboid morphologies and persisted in the fissure, significant differences between IL and CL hippocampus were observed (Figure 11). At 24 hours, increased Iba-1+ macrophages were observed in the third ventricle, and the microglial response was maintained in the hippocampal fissure (Figure 9(h)), but Iba-1+ round-shaped microglia/macrophages started to cover the degenerating CA fields (Figure 9(h)). Notably, at this time, although morphological and distribution changes in the microglial response versus the contralateral hippocampus were evident (Figure 9, compare 9(e) and 9(h)), the area occupied by reactive microglial cells did not differ significantly from the contralateral side (Figure 11), probably as a consequence of the reduced total cell area of pseudopodic/ameboid cells versus ramified cells. From 48 hours to 7 days after hypoxia, a massive increase in microglia/macrophage cell intensity was evident in the fissure and CA field (Figures 9(i), 9(j), 9(l)–9(n)), showing a 5–7-fold increase in the area occupied by reactive microglia/macrophages when compared to the contralateral hippocampus (Figure 11). At longer survival times, microglial response was strongly decreased, showing scattered reactive ramified and macrophages in the fissure and CA only until 14 days (Figures 9(o), 9(s), and 9(t)), but no presence of reactive microglia/macrophages at 30 and 100 days (Figures 9(p), 9(q)). It should be noted that only scattered activated microglial cells were present in the DG, and always located in the hilus, correlating with the above described astroglial response in this area which is mostly spared in this neonatal injury model as a consequence of its late development [53, 54].

**Corpus Callosum**

The corpus callosum, like other white tracts including the internal and external capsules, showed microglial response characterized by the presence of reactive ramified cells elongated in parallel to axonal tracts, from a few hours after the insult (Figure 9(g)), and some ameboid microglia/macrophages observed at 24–72 hours after hypoxia (Figures 9(h)–9(k)) and until 7 days (Figure 9(n)), when response diminished (Figures 9(o) and 9(r)), almost returning to basal level at 14 days after hypoxia. However, it should be noted that in this region only mild differences in relation to the contralateral side were seen, with no statistically significant differences shown in the Iba-1+ area at any timepoint (Figure 11), This pattern of microglial response in the ipsilateral versus contralateral white matter correlated with the mild response of astroglial cells described above although the changes in glial cells of the contralateral corpus callosum, which also results mildly atrophied, may be masking the increases in glial response in the ipsilateral side.

**Caudate-Putamen, Neocortex, and Thalamus**

In general, in these areas, microglial response was also seen as early as 3 hours after hypoxia and lasted until 7 days although it showed a high degree of variability and very few significant differences in compared to the contralateral hemisphere (Figure 11). Reactive microglial cells mainly showed an activated ramified morphology and increased Iba-1 labeling (Figures 10(d)–10(f) and 10(j)–10(l)) although some pseudopodic/amoeboid microglial cells were seen from 12 to 72 hours after lesion, when maximum responses were seen (Figures 10(e) and 10(k)). In the caudate-putamen, Iba-1+ cells have shown the higher activation in the ventral-lateral region. At 7–14 days after hypoxia, in all three regions, microglial response remained as patches of reactive ramified microglial cells (Figures 10(f) and 10(l)).

### 3.4. Neutrophil Recruitment

#### 3.4.1. Distribution of Neutrophils in the Control Postnatal Brain

**Figure 12:**
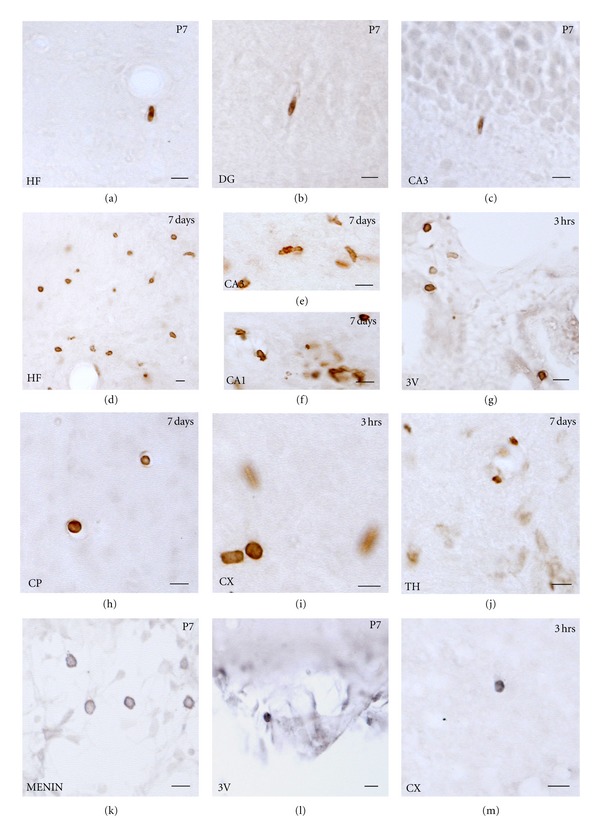
Leukocyte infiltration is monitored by analysing neutrophils (Ly6B2 staining) and T-lymphocytes (CD3 staining). Ly6B2 staining in the control animals at P7 shows a few neutrophils inside the blood vessels in the hippocampal fissure (a), in DG (b), and in the CA (c) regions. In the hippocampus the maximum density of cells is observed at 7 days after hypoxia especially in the hippocampal fissure (d); CA3 (e) and CA1 (f). In caudate-putamen an increase is observed at 7 days after hypoxia (h). In the ventricles (g) and the neocortex (i) the maximum quantity of neutrophils is observed at 3 hours after hypoxia. No significant difference was observed in the thalamus (j). CD3 staining in the control animals shows the presence of lymphocytes in the meninges (k) and in the ventricles (l). Finally, scattered cells are observed in the neocortex (m). Scale bars: 10 *μ*m. Hf: hippocampal fissure; DG: Dentate gyrus; CA: cornu ammunis.

Neutrophils were generally not present in control brain parenchyma. Only scattered neutrophils were seen in the medial or lateral third ventricle at P7–P21, in decreasing numbers with hardly countable cells at P21. At these ages we also observed a few cells in blood vessels located in hippocampus and neocortex of both hemispheres (Figures 12(a)–12(c)). Scattered neutrophils were also seen in the meninges/median fissure. In comparison to adults, neonates are known to have weakened neutrophil response and reduced tendency to extravasate from blood vessels [55–57].

#### 3.4.2. Distribution of Neutrophils in the Contralateral Hypoxic Hemisphere

**Figure 13:**
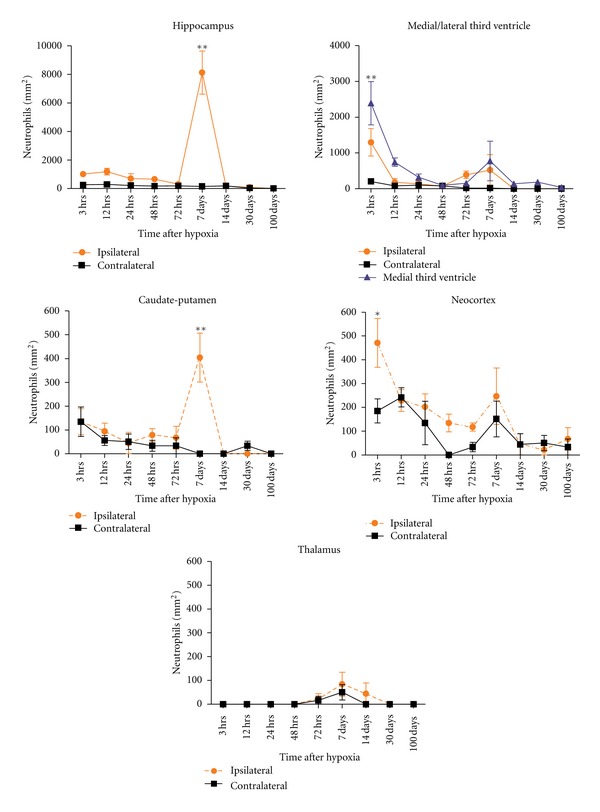
Neutrophils quantification is done using image J software (NIH) and is evaluated at 3–72 hours and 7–100 days. The regions analysed were the hippocampus, medial/lateral third ventricle, caudate-putamen, neocortex, and thalamus. The quantity in the ipsilateral side is compared to the contralateral hemisphere. Significant changes are observed in the ipsilateral side of the hippocampus and caudate-putamen at 7 days after hypoxia while a decrease in number is observed after 3 hours in the ventricles and neocortex. There is no change in the thalamus. All values are represented as mean ± S.E.M and are corrected using Abercrombie correction method. Two-way ANOVA with Bonferroni post-hoc analysis is used to compare ipsilateral versus contralateral hemisphere at all time points and *P < 0.01, **P < 0.001 was considered significant.

At 3 and 12 hours after hypoxia, some neutrophils were observed inside the blood vessels in the neocortex, caudate-putamen, and in the hippocampus, but also in the lateral side of third ventricle. By 24–72 hours, neutrophil cell numbers decreased in the blood vessels of neocortex and in the third ventricle (Figure 13). At 7–14 days after hypoxia, some neutrophils were observed in the medial third ventricle (Figure 13), the neocortex, and the thalamus. At 30 and 100 days after injury, there was hardly any cell found in the brain blood vessels or the parenchyma (Figure 13).

#### 3.4.3. Distribution of Neutrophils in the Ipsilateral Hypoxic/Ischemic Hemisphere

**Hippocampus**

Neutrophils were observed in the ipsilateral hippocampus as early as 3 hours after hypoxia (Figure 13). Cells were usually found distributed in the hippocampal fissure, the dentate gyrus, or the fimbria. At 12 hours after hypoxia, the number of cells increased and was localised in the CA3 region, in the parenchyma as well as inside the blood vessels. In the hippocampal fissure, the dentate gyrus and in the fimbria, most of the neutrophils were inside the blood vessels (Figure 13). At 24 hours after hypoxia, neutrophils were observed throughout the hippocampus but mainly localised in CA1 region and the fimbria (Figure 13). By 48 hours after hypoxia, neutrophils were not observed in the dentate gyrus though a few cells were present near CA3 and the fimbria (Figure 13). Neutrophils appeared to be evenly distributed throughout the hippocampus after 72 hours after hypoxia, but significantly higher density of cells were observed at 7 days after hypoxia (Figures 12(d)–12(f) and 13). At this time of maximum neutrophil numbers, the cells were mostly observed near the hippocampal fissure, CA1 and CA3 region, with the majority of cells in the parenchyma, but usually concentrated near the blood vessels (Figures 12(e) and 12(f)). At 14 days after hypoxia, the amount of cells rapidly decreased although a few cells were still found, in close opposition to blood vessels in the hippocampal fissure and around the CA3 region. At 30 days after hypoxia, very few neutrophils inside the blood vessels could be identified, and at 100 days after hypoxia no neutrophils were seen inside the hippocampus.

**Ventricles**

An elevated number of cells were also present in the third ventricle, both medially and in the ipsilateral side of the third ventricle as early as 3 hours after hypoxia (Figures 12(g) and 13). At 12–48 hours, the quantity of cells progressively decreased, but they were mostly distributed in the medial part (Figure 13). By 7 days after hypoxia, correlating with increased numbers also in hippocampus, an increase in neutrophils both in the medial and ipsilateral side of the ventricle could be seen (Figure 13). Finally, by 14 to 100 days, no neutrophils were seen in the lateral side of the third ventricle although scattered cells were located in the medial part.

**Caudate-Putamen, Neocortex, and Thalamus**

From 3 hours to 72 hours, only a few neutrophils were located in the caudate-putamen region (Figure 13). An increase in the number of cells was seen at 7 days (Figures 12(h) and 13), correlating with previously described areas. At longer survival times, no neutrophils were seen in this region.

At 3 hours after hypoxia, some neutrophils were distributed in the blood vessels of different layers of the neocortex (Figure 12(i)), being the time showing the highest density (Figure 13). From 12 to 72 hours a reduction in neutrophil cell counts was generally observed although by 72 hours a few cells remained in the upper layers of neocortex. At 7 days after hypoxia, there was a mild increase in neutrophils located inside the cortical blood vessels in both hemispheres. At 30 and 100 days, almost no neutrophils were present in the neocortex, and if so, they were located inside the blood vessels (Figure 13).

In the thalamus, very few cells were observed as compared to the other regions analysed. No neutrophils were observed from 3 to 48 hours after hypoxia, and only a few cells were seen at 72 hours, 7 and 14 days (Figures 12(j) and 13). From 30 days, neutrophils were no longer present in the thalamus (Figure 13).

### 3.5. Lymphocyte Distribution in the Control and the Hypoxic/Ischemic Brain

In the control brain and at all ages analysed, scattered lymphocytes were only located in the ventricles and meninges, although scattered single cells were sometimes seen in the hippocampus, neocortex, always inside the blood vessels (Figures 12(k), 12(l) and 12(m)). At all time points analysed after hypoxia, no changes were seen in the contralateral or the ipsilateral hemisphere when compared to control.

## 4. Discussion

In this study we have performed a detailed short and long-term analysis of neuropathological changes, astroglial, microglial response, and leukocyte recruitment following H/I to the neonatal mouse brain, describing massive damage and cellular changes in the ipsilateral hemisphere, but also not negligible changes in the contralateral side. Several of these results will be discussed in separate sections.

### 4.1. Neuropathological Changes in the Ipsilateral H/I Hemisphere

Our description of neuropathological changes in the ipsilateral hemisphere is in agreement with previous reports [18, 42, 58, 59], showing hippocampal damage as the most striking feature of hypoxic/ischemic damage in the neonatal mouse, whereas damage to caudate-putamen, neocortex, and thalamus is highly dependent on the postnatal age and the duration of the hypoxia. Hippocampal damage with tissue disruption, neuronal damage, and disorganization of the CA cytoarchitecture was observed as early as 3 hours after hypoxia followed by milder damage to DG at later survival times, which is maintained relatively spared due to its postnatal development. At 7 days after hypoxia, significant atrophy of hippocampal area is evident. This temporal pattern of neurodegeneration is consistent with the observation from other studies showing that H/I damage in an immature brain evolves more rapidly than its adult counterpart [40, 60].

We observe subcortical white matter damage and long-term atrophy, which has been described as a hallmark of neonatal H/I in preterm infants, where the oligodendrocytes in the periventricular white matter are considered one of the most vulnerable cell types to H/I damage [61, 62]. In rodent models, neonatal H/I injury has been shown to cause axonal degeneration [63] and disturbances in myelination [64, 65]. Following H/I in the P9 mouse, several authors have reported decreased expression levels of myelin basic protein (MBP) and proteolipid protein (PLP), decreased neurofilament expression, and the presence of apoptotic cells in the corpus callosum within 24 to 72 hours after injury [66, 67]. White matter damage has been related to the loss of immature oligodendrocytes in the tracts as well as the loss of subventricular zone (SVZ) progenitors after H/I, inducing a depletion of oligodendrocyte precursors [68, 69].

Another area showing consistent damage and atrophy in the mouse model of H/I is the caudate-putamen, and the neocortex to a lesser extent and showing higher variability. In this regard, a recent study by Selip and coworkers using the neonatal rat model [70] have shown that rats with moderate or severe loss of MBP having significantly increased axonal degeneration in the temporal-parietal cortex, caudate-putamen, thalamus, and internal capsule. Moreover, pups without evidence of severe white matter loss exhibited mild selective grey matter injury, as evidenced by mild axonal injury and neuronal degeneration, in the cortex, internal capsule, and caudate-putamen; structures central to language processing and understanding, and motor and sensory function. Injury in these regions, even if mild, may be implicated in the neurocognitive disturbances noted in preterm survivors who do not demonstrate other clinical or radiological evidence of overt periventricular white matter injury [71]. It is interesting to note that we here describe in the mouse that caudate-putamen and cortical atrophy are mainly noted as a long-term effect but show very disperse injury scores at early survival times.

### 4.2. Contralateral Hippocampus and Corpus Callosum Show Mild Long-Term Atrophy

Moreover, the effect of H/I in the contralateral hemisphere has been studied extensively to suggest that it cannot be used as an efficient control for histological assessment of brain damage in mice, in contrast to what has been described previously in the neonatal rat [60, 72, 73], providing an significant difference in these species response to H/I.

Previous studies using the rat model of H/I have demonstrated that the blood flow to the contralateral cerebral hemisphere structures is relatively unchanged during hypoxia [74], and that the contralateral hemisphere, when evaluated several weeks after the injury, shows no tissue alterations or atrophy, suggesting that the contralateral hemisphere can be used as a “control” reference for the evaluation of the extent of damage in the ipsilateral hemisphere in the rat [73]. Some molecular changes in kinases and proinflammatory molecules have been described in both hemispheres in H/I neonatal rats [75, 76]. Also Jansen and Low [72] histologically assessed a hypertrophy of the contralateral hemisphere in adult rats that had undergone perinatal H/I. In the mouse brain, several laboratories have shown that it does not suffer apparent changes during the first week following H/I [18] as is also commonly used as a reference to evaluate the ipsilateral hemisphere. Interestingly, inflammatory gene profiling in P9 mouse brain after H/I shows more than 140 genes involved in the tissue response during the first 72 hours; however, only microglial expression of osteopontin showed an increase in contralateral subcortical white matter [77]. While mice and rats show distinguished regional features in tissue damages, it would be interesting to analyze the molecular changes in HI-neonatal mice. Nevertheless, it should be noted that we here report that analysis of the H/I mouse brain up to 3 months after the injury shows some degree of atrophy in the contralateral hippocampus and the corpus callosum, accompanied by ventricle swelling, an event that has been reported earlier [78].

The immature brain has a tendency of considerable compensatory reorganization following injury. There are reports stating compensatory reorganizational changes occurring in the contralateral hemisphere in some animals following neonatal H/I brain injury and that this plasticity may be functionally advantageous [72]. Moreover, the presence of significant cognitive deficit in apparent unilateral focal brain injury also indicates towards the involvement of contralateral hemisphere [79]. In this sense, it should be noted that H/I animals undergo systemic hypoxia, which has been shown to induce changes in gene expression and cell activity by itself. As an example, change in the expression of certain cytokines, like Hypoxia Inducing Factor alpha (HIF*α*), and P-Akt to the same extent in both the ipsi—as well as contralateral hemisphere showed that hypoxia is sufficient to regulate multiple mediators that may contribute, but may not be sufficient to induce long-term neuronal damage [76].

### 4.3. Glial Response

#### 4.3.1. Transient Astroglial Response in the Contralateral Hippocampus and White Matter and Long-Term Glial Scar Formation in the Ipsilateral Hemisphere

As reviewed by Sofroniew and Vinters [80], many gray matter astrocytes in healthy CNS do not express GFAP at immunohistochemically detectable levels or express low levels as in neonates. In our immunohistochemically processed sections, although GFAP expression was seen in control neonatal brains, an increase in GFAP immunostaining was observed after H/I from early time points (3–12 hours) in comparison to P7 age-matched control, implying an onset of astrogliosis.

Notably, changes in astroglial morphology by GFAP immunostaining were first seen at 3 hours post-hypoxia both in the ipsilateral as well as contralateral hemisphere. In the contralateral hemisphere, increased GFAP and astroglial hypertrophy showed a maximum response at 24–48 hours after hypoxia but decreased at longer survival times. In the contralateral side, astroglial response was very restricted to the corpus callosum and the area of the hippocampal fissure, but never covering the CA-neuronal layer. However, in the ipsilateral H/I-damaged hemisphere, the increase in GFAP expression and cell hypertrophy peaked at 14 days after hypoxia and was evident in the corpus callosum, the caudate-putamen, the neocortex, and the hippocampus, where the long-term glial scar persisted till 100 days after hypoxia.

As the radial glia mature, they show GFAP expression and some give rise to GFAP-expressing radial neural stem cells (NSCs) that persist in juvenile and adult forebrain, while others become astrocytes [81–83]. Some of these radial NSCs remain constitutively active throughout life in the subventricular zone of the lateral ventricles and in the subgranular zone of the hippocampal dentate gyrus, where they are the predominant source of adult neurogenesis. This might be the reason of concentration and persistence of glial scar or GFAP+ cells at 14–100 days after hypoxia in hilus and hippocampal fissure in our study. Notably, GFAP expression after H/I was also found most highly concentrated in layers showing high content of synaptic contacts including the hippocampal fissure in the neonatal brain as seen in our study, which is in concordance with reports where astrocytes appear to influence developmental synaptic pruning by releasing signals and thereby tag them for elimination by microglia [84, 85].

The role of reactive astrogliosis in the evolution of ischemic brain lesions especially in neonates is at present not clear, but recent studies have suggested that reactive astrocytes provide essential metabolic support to neurons during transient ischemia and that failure of astrocyte functions may contribute to neuronal degeneration [86, 87]. Additionally, in adult transgenic mice, experimental disruption of astroglial scar formation following stroke is associated with loss of barrier functions along the margins of infarcts, resulting in increased spread of inflammation and increased lesion volume [88]. Moreover, adult mice lacking GFAP [GFAP(−/−)] show attenuated reactive gliosis, reduced glial scar formation after focal brain ischemia as compared to injured developing brain where there is only an increase in the survival of newborn neurons [89].

Astrocytes also play a vital role in white matter, regulating molecules such as glutamate in the extracellular space and preventing excitotoxic damage to neighbouring oligodendrocytes and axons. GFAP knockout mice exhibit degeneration of myelin with progressing age [90]. Consistent with previous reports, we noted an increase in GFAP expression in white matter astrocytes accompanied by hypertrophy and process thickening in ipsilateral hemispheres [91].

#### 4.3.2. Transient Microglial Response in the Contralateral Hemisphere and Widespread in the H/I Damaged Side

In control postnatal mice, we observed amoeboid and ramified microglia throughout gray and white matter from P7 to P14 mice, as has been described previously [92]. As the brain development continues after birth, microglial cells need to adapt to the changes in the microenvironment [92]. Until P14, we observed groups of amoeboid microglial cells which are present in the developing corpus callosum, cingulum, and fimbria. These cells are proposed to be involved in the phagocytosis of cellular debris and contribute to the axonal nerve fiber remodeling and synapsis during normal development [93–95].

 In the present study, we have observed morphologically activated microglia from 3 to 72 hours after hypoxia in the contralateral corpus callosum, with a peak of response at 24 hours. This microglial response to hypoxic conditions in the subcortical white matter has been extensively studied by the group of Ling and coworkers, who have demonstrated that hypoxia-activated microglial cells in the developing white matter produce several inflammatory mediators including cytokines, chemokines, and reactive oxygen species which are detrimental for white matter development and oligodendrocyte survival (reviewed in [96]), which may account for the long-term contralateral corpus callosum atrophy we observe, although the microglial response in the contralateral corpus callosum is transient, in agreement with the findings of Zaidi and coworkers [97] in the P7 rat model, that did not observe activated microglia after 14 days of hypoxia in the contralateral hemisphere. Interestingly, in agreement with our observations, Cowell and coworkers [98] have shown a transient contralateral microglia activation in the cortex, white matter and hippocampus after an unilateral transection of MCA in neonatal rat brain.

Obviously, microglial response in the ipsilaterally damaged corpus callosum is very striking, showing reactive ramified and ameboid/macrophagic forms from 3 hours to 14 days after hypoxia, with a peak of response at 48–72 hours. It is now evident that the developing brain is highly susceptible to hypoxic damage because of its high oxygen and energy requirements [99, 100], and that white matter at this developmental stage is vulnerable. Moreover it have been described that the myelin from the degenerating axons is phagocytosed by microglia [101]. In this sense, as long-term atrophied white matter is observed after microglia returns to a resting state, we may suggest that activated microglial cells may not be sufficient to complete phagocytosis and avoid the inhibition of oligodendrocyte precursors differentiation. As most of this knowledge is mainly obtained from results in rat models and several differences has been described between rodents, a more detailed description on the late effects on oligodendrocytes, their precursors, myelination and axonal degeneration in neonatal mice brain hypoxic ischemic injury is needed.

Interestingly, microglial response in the contralateral gray matter areas was more evident that the astroglial response, and activated microglial cells were seen as early as 3 hours after hypoxia in the hippocampus, but also in the caudate-putamen and cortex (see Figures 9 and 10); however, contralateral microgliosis was very transient and only persisted until 48–72 hours depending on the regions. In the contralateral hippocampus, microglial response was mostly evident in the hippocampal fissure, and not so widespread as in the ipsilaterally damaged side, where we describe a layer-specific activation of microglia as early as 3 hours after hypoxia, with a maximum response from 48 hours to 7 days, followed by a patchy pattern at later time points. This has also been demonstrated at early time points in rat model as mentioned previously by Cowell and coworkers [98]. Remarkably, hippocampal microglial response was first observed surrounding the blood vessels in the hippocampal fissure, which have been suggested to be more vulnerable to ischemic episodes than those from other hippocampal areas [102]. Interestingly, this is known to be one of the sources of microglia progenitors during late embryonic life in the rat, showing, during early postnatal development an outside-to-inside microglia distribution pattern towards the pyramidal or granular cell layers [92]. From 24 hours onwards evident neuronal damage when evident neuronal damage takes place in the ipsilateral hippocampus and then ameboid/macrophagic phagocytic microglia populate the neurodegenerating CA areas.

 The association between microglia activation and injury development raises the question whether this reaction is detrimental or beneficial [103–105]. Traditionally, microglia activation was considered harmful [19, 58]. However, it is now established that, as macrophages do in the periphery, microglia has two different patterns of activation and function in response to CNS injury (revised by [26, 103, 106]). Then, selective ablation of proliferating microglial cells exacerbates ischemic injury [107]. Moreover, opposite effects have been described in neonatal H/I mice and rats using minocycline, a tetracycline derivative that nonspecifically blocks all microglia activation. In rat brain, this treatment protects the brain tissue in some reports [19, 20] but only have a transient protective effect in others [108]. In contrast, tissue damaged increases in minocycline-treated H/I mice, especially in cortex, caudate-putamen and thalamus without significant effects on hippocampus [20]. Additionally, selective depletion of microglia before a transient MCAO in a P7 rats does not change the volume of injury but enhances cytokines production compared to not depleted animals [109], suggesting a beneficial role of microglial cells.

These evidences made a complete characterization of neonatal mice microglial response essential, in order to define the better window and target for protective therapies. New insides in the physiological activity of microglia (called “surveillance” instead of “resting”), joined to adult MRI and behaviour assessment [78, 110], would be beneficial to promote phagocytic and anti-inflammatory response of microglia than a complete blocking of their activation in order to obtain better outcomes of therapies applied to injured developing brain.

#### 4.3.3. H/I Induces Neutrophil Recruitment but Very Low Presence of Lymphocytes

 The neonates are known to have weakened neutrophil response and reduced tendency to leukocyte extravasation from blood vessels [55–57]. Previous studies have demonstrated that neutrophils contribute to the long-term hypoxic/ischemic brain injury in the neonatal rat brain [111, 112]. We here report that neutrophils appeared as early as 3 hours after hypoxia in blood vessels of most of the regions studied, especially in the neocortex and third ventricle, in agreement with previous reports showing that neutrophils are seen in brain blood vessels rather early [111, 113, 114]. However, there are limited studies reporting neutrophils in the neonatal parenchyma after hypoxia, and the results are variable; we observed neutrophil recruitment to the injured mouse parenchyma (mainly hippocampus and caudate-putamen) after 72 hours to 7 days after hypoxia, whereas other studies have shown neutrophils accumulated in the injured rat parenchyma at 12–24 hours after hypoxia, peaking at 72–96 hours [113, 115]. Notably, neutrophils accumulate in the same areas of microglia/macrophage accumulation, contributing in the removal of cellular debris and the release of cytokines to further attract more immune cells to the injury site [114, 116]. The negligible lymphocytic infiltration reported here is in accordance with previous reports where no CD3+ cells were detected in the neonatal P1 rat brain at 48 hours after hypoxia and LPS induction [117]. Furthermore, there are reports of very low expression of CD3*γ* chain of the T-cell receptor in P3, P7, and P14 mice brain in contrast to adult [33].

Since many investigators are using transgenic and knockout mice to determine the importance of specific molecules in the evolution of damage after brain injury, there is an urgent need to perform comparative studies on the relative vulnerability of the mouse brain in comparison to other species. A mouse model of hypoxic-ischemic encephalopathy has paved a way for the description of the specific molecular mechanisms associated with this destructive disease, by the use of genetically modified animals. Our major finding describing the short- and long-term effects as well as the involvement of the contralateral hemisphere may serve as a valuable resource for functional definition of neuroprotection or damage as well as will aid in selecting the time and mode of intervention in the broad therapeutic window.

## 5. Conclusion

To summarize, this study describes qualitatively and quantitatively the tissue damage, glial response, and inflammatory cell recruitment after brain injury induced by carotid occlusion and systemic hypoxia (8% O2, 55 minutes) to the postnatal day 7 mouse brain, analyzing changes from 3 hours to 100 days after hypoxia. In general, massive tissue injury and atrophy in the ipsilateral hippocampus, corpus callosum and caudate-putamen are consistently shown, with neutrophil recruitment and earlier microgliosis, but persistent long-term glial scarring until 100 days after hypoxia. Remarkably, in the contralateral hippocampus and corpus callosum, milder atrophy is delayed in areas that show the activation of astrocytes and microglial during the first 72 hours. This study highlights that care should be taken when using the contralateral hemisphere as control while studying ipsilateral H/I injury in postnatal mouse brain.

## Figures and Tables

**Table 1:** Injury score grading system. Survival times from 3 to 72 hours after hypoxia.

Hippocampal CA field
(0) No damage
(1) Only one/two patches of neurodegeneration
(2) More than 3 neurodegeneration patches
(3) Most CA1 or CA3 damaged
(4) All CA1 and CA1 damaged

Hippocampal DG

(0) No damage
(1) <40% of DG neurons damaged
(2) Approximately 50% of DG neurons damaged
(3) >60% of DG neurons damaged

Corpus callosum

(0) No changes seen
(1) Increased cellularity in ipsilateral corpus callosum
(2) Increased cellularity in ipsilateral corpus callosum and
swelling

Caudate-Putamen

(0) No damage
(1) <40% of striatal area damaged (usually with increased
cellularity in white matter patches)
(2) Approximately 50% of striatal area damaged
(3) >60% of striatal area damaged

Neocortex

(0) No damage
(1) Scattered neurodegeneration columns in cortex
(2) Neurodegeneration columns in most cortical areas
(3) General neurodegeneration in several areas, all layers

Thalamus

(0) No damage
(1) <40% of thalamic area damaged (only rostral thalamus)
(2) Approximately 50% of thalamic area damaged
(3) >60% of thalamic area damaged
(extending to caudal thalamus)

**Table 2:** Injury score grading system. Survival times from 7 to 100 days after hypoxia.

Neuronal density in hippocampal CA fields
(0) No reduction
(1) More than 60% of CA neurons remaining
(2) Approximately 50% of CA neurons remaining
(3) Between 10 and 40% of CA neurons remaining
(4) Less than 10% of CA neurons remaining

Hippocampal CA field

(0) No damage
(1) Only one/two patches of neurodegeneration
(2) More than 3 neurodegeneration patches
(3) Most CA1 or CA3 damaged
(4) All CA1 and CA1 damaged

Neuronal density in hippocampal DG

(0) No reduction
(1) More than 60% of DG neurons present
(2) Approximately 50% of DG neurons present
(3) Between 10 and 40% of DG neurons present
(4) Less than 10% of CA neurons remaining

Corpus callosum atrophy

(0) No reduction
(1) More than 60% of tissue remaining (less than 40% atrophy)
(2) Approximately 50% of tissue remaining
(3) Less than 40% of tissue remaining (more than 60% atrophy)

Corpus callosum cellularity

(0) No changes seen
(1) Increased cellularity in corpus callosum
(2) Increased cellularity in corpus callosum and swelling

Caudate-Putamen atrophy

(0) No reduction
(1) More than 60% of tissue remaining (less than 40% atrophy)
(2) Approximately 50% of tissue remaining
(3) Less than 40% of tissue remaining (more than 60% atrophy)

Neocortical atrophy

(0) No reduction
(1) More than 60% of tissue remaining (less than 40% atrophy)
(2) Approximately 50% of tissue remaining
(3) Less than 40% of tissue remaining (more than 60% atrophy)

Thalamic atrophy

(0) No reduction
(1) More than 60% of tissue remaining (less than 40% atrophy)
(2) Approximately 50% of tissue remaining
(3) Less than 40% of tissue remaining (more than 60% atrophy)
